# The American Medical Association

**Published:** 1877-07-20

**Authors:** 


THE AMERICAN MEDICAL ASSOCIATION.—This body, at its late meeting, refused to repeal or modify their previous action in regard to the excommunication of the Arkansas State Medical Assoeiation. It may reasonably be expected that this will prove an entering wedge to much future division, discord, and bitterness in the profession.

